# Dialkylketones in Paperboard Food Contact Materials—Method of Analysis in Fatty Foods and Comparative Migration into Liquid Simulants Versus Foodstuffs

**DOI:** 10.3390/molecules25040915

**Published:** 2020-02-18

**Authors:** Antía Lestido-Cardama, Ángela Störmer, Roland Franz

**Affiliations:** 1 Department of Analytical Chemistry, Nutrition and Food Science, Faculty of Pharmacy, University of Santiago de Compostela, 15782 Santiago de Compostela, Spain; 2 Fraunhofer Institute for Process Engineering and Packaging (IVV), Giggenhauser Straβe 35, 85354 Freising, Germany; angela.stoermer@ivv.fraunhofer.de (Á.S.); roland.franz@ivv.fraunhofer.de (R.F.)

**Keywords:** dialkylketones, alkylketene dimers (AKD), paperboard, food contact material, migration, fatty food, olive oil, cheese, NIAS, GC-FID

## Abstract

Dialkyl diketene dimers are used as sizing agents in the manufacture of paper and board for food contact applications to increase wetting stability. Unbound residues can hydrolyze and decarboxylate into dialkylketones. These non-intentionally added substances (NIAS) have potential to migrate to fatty foods in contact with those packaging materials. In Germany, the Federal Institute for Risk Assessment (BfR) established a specific migration limit (SML) of 5 mg/kg for the transfer of these dialkylketones into foodstuffs. In order to investigate the differences between simulants and real foods, an analytical method was optimized for extraction and quantification of dialkylketones in edible oils and fatty foods by gas chromatography coupled with flame ionization detection (GC-FID), and additionally by gas chromatography with mass spectrometry (GC-MS), to confirm their identification and to quantify them in case of interferences. Dialkylketones are separated from the extracted fat by alkaline saponification of the triglycerides. Dialkylketones migration from paper-based food contact articles into organic solvents isooctane and dichloromethane, in olive and sunflower oils, and in fatty foods (croissants, Gouda, cheddar cheese, and salami was studied). As a result, it was found that the simulating tests, including the edible oil extraction tests, gave migration values that exceeded the SML largely, while the migration with the food samples were largely below the SML.

## 1. Introduction

While migration from (and safety of) plastic materials in contact with food has been studied extensively for several decades, and plastic food contact materials (FCM) are subject to specific EU legislation (no 10/2011), research on migration from fiber-based FCMs is still in its infancy. Furthermore and, possibly as a consequence, so far there has been no specific EU-wide harmonized regulation on paper and board (P&B) FCM. Instead, on a national level, certain regulations do exist, such as the internationally recognized Recommendation XXXVI for paper and board, intended for food contact of the German Federal Institute for Risk Assessment (BfR). These recommendations are based on scientific evidence, where available, as well as largely on conventions, due to a lack of sufficient scientific data concerning migration under realistic conditions; hence, exposure from P&B FCM constituents. Such data and knowledge are indispensable as a basis for future legislation and rules for the paper packaging industry.

Less than 3.5% of all food packaging in direct contact with food are made from uncoated and untreated paper and board; thus, consumer exposure is estimated to be equally low. In addition, the foods that are in direct contact with this type of material are mainly dry foods (approximately 50%) and foods that need to be peeled or washed before their consumption (approximately 30%), so in these cases, the migration of substances is also expected to be low. [1]. One of the critical properties of uncoated and untreated P&B for food packaging applications is its low resistance to high moisture content, which can cause disintegration of the P&B material in such applications [1,2]. To overcome this, technical solutions are the use of coatings or plastic layers for direct contact with food [1]. Another solution could be hydrophobization of the fibers by introducing chemicals as additives into the pulp stock. This phenomenon, known as sizing of paper, allows improving its capacity to resist a certain degree on wetting with liquids, besides improving their dimensional stability and the quality of the surface [1,2,3].

There are two main methods for applications of different conventional sizing: to add it in the paper machine at the wet end, known as internal sizing, or using a size press, which is surface sizing [2]. The main internal sizing chemicals currently in use around the world are based on emulsions of rosin, alkyl ketene dimer (AKD), and alkenyl succinic anhydride (ASA). In all three cases, the objective is establishing a barrier against penetration and spreading of the liquid through the porous structure of the paper by protecting the hydroxyl groups in the sheet of the paper. This also improves the printing capacity of the paper and reduces the effect of aqueous fountain solution on loss of paper strength [3].

AKD has become the most widely used internal sizing agent in the world since it was introduced to the papermaking industry in the 1950s due to its high efficiency and ease of use [4,5,6]. Commercial AKDs are prepared from natural fatty acid sources; the stearic acid is mainly used for this purpose. Therefore, chemically, AKD sizing agents are typically mixtures with varying chain lengths according to the fatty acid fraction used for the synthesis. [7].

**Figure 1:**
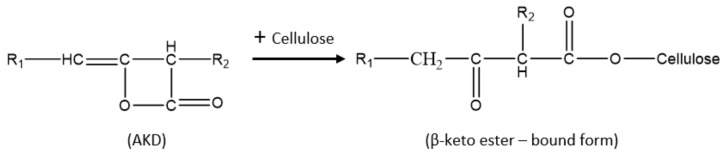
Reaction of alkyl ketene dimer (AKD) with cellulose.

**Figure 2:**
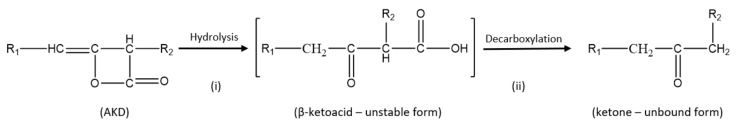
Hydrolysis of AKD (i) followed by decarboxylation of AKD (ii).

In general, it is assumed that the sizing mechanism of AKD involves the reaction of its β-lactone ring with the hydroxyl groups of cellulose molecule to form a covalent β-keto ester bond (Figure 1); hence, making the paper resistant to liquids. A study by Lindström and Söderberg (1986) [8] showed that the sizing effect depends mainly on the quantity of chemically reacted (bound) AKD in paper. However, some AKD may undergo hydrolysis in the presence of water molecules producing an unstable β-keto acid, which spontaneously decarboxylates to form the corresponding dialkylketones (DAK) (Figure 2). This portion of AKD remains non-reacted, in the sense of not bound to cellulose and its reaction products, the DAK, absorb into the fibers. AKD molecules may also react together to form oligomers [4].

Consequently, these DAK are present in many P&B-based food contact materials and are introduced in the recycling loop. DAK were identified, for instance, in extracts of recycled paper products and in the isooctane extract of a hot-cup starch lined by gas chromatography with mass spectrometry (GC-MS) [9,10]. They can be considered as a non-intentionally added substance (NIAS) to the packaging, since they originate from AKD degradation processes. These AKD based DAK molecules are characterized by a central carbonyl group and two long aliphatic lateral chains whose length depends on the original fatty acids involved in decarboxylation process. These two long aliphatic chains make the DAK highly lipophilic, similar as linear alkanes with the same C number. Their nomenclature refers to the total carbon atoms present in the two lateral aliphatic chains, minus the one lost in the decarboxylation process, together with the numerical position of the carbonyl group [11]. Accordingly, the DAK with two stearyl chains (C17COC17) is named ‘stearone’ with an estimated log Po/w = 16 [12]. Based on the typical average molecular weight of dialkylketones (for stearone: m.w. = 507 g/mol), their volatility is quite low. Consequently, their migration into food will take place only in case of intimate contact with the food, or will strongly depend on the temperature in case of indirect contact through the gas phase.

From a regulatory point of view in Europe, the German Federal Institute for Risk Assessment (BfR) allows the use of AKD as sizing agents for the manufacture of paper and board intended to come into contact with foodstuffs, in accordance with the Recommendation XXXVI “Paper and board for food contact”. However, in order to protect the consumer´s health, the following provision is established there: “Di-alkyl(C10-C22)diketenes, which can contain up to 65% isoalkyl groups, max 1.0%. The transfer of dialkylketones, that are produced by hydrolysis, into foodstuff may not exceed 5 mg/kg foodstuff” [13]. The Food and Drug Administration (FDA) of the United States, in its Code of Federal Regulations (CFR) title 21, part 176.120, allows the use the alkyl ketene dimers as an adjuvant in the manufacture of paper and paperboard, but with the condition that the alkyl ketene dimers and their hydrolysis products dialkylketones do not exceed 0.4 percent by weight of the paper and paperboard [14]. To control these restrictions, in particular the BfR specific migration limit of 5 ppm, the contact of paper and board with fatty food matrices must be especially taken into consideration due to the high lipophilicity and, hence, fat solubility of DAK. Liquid simulants, such as vegetable oils or isooctane, soak the P&B samples during contact and act, therefore, more or less extractive. The main packaging application of this type of P&B materials is (semi)solid foods. For liquid contact, coated or otherwise impregnated P&B materials would be needed. Solid or semi-solid real fatty foods, such as cheese, sausages, or fatty bakery products, show a different interaction with the P&B; however, migration data on real foods are not available. The 95% ethanol showed lower transfer than olive oil and isooctane [15], which can be explained by lower solubility of the DAK in ethanol.

Interestingly, DAK molecules are not only relevant as potential migrants from P&B FCM, but were also identified as a new important class of compounds, newly found in the unsaponifiable fraction of vegetable oils that had undergone interesterification [11]. In those processes, DAK are formed as by-products. Interesterification of fats is a common industrial practice in order to redistribute fatty acids in triglycerides under the influence of a chemical catalyst or using an enzyme [16]. Those fats are used e.g., in margarine, chocolate, and bakery products [11,17]. It should be noted that the total DAK content determined in interesterified coconut oil, shea oil, and palm oil samples ranged between 80 and 2900 ppm [11].

Analytical methods to determine the DAK in paper samples are summarized in [3]. Analytical methods for quantification of DAK in oil are published by Derra and Jung [15] and Santoro et al. [11].

The two major objectives of this work were: (i) to establish a convenient sample work up-procedure and an analytical method based on the methodology published by Santoro et al. [11] for the determination of DAK in fatty foodstuffs. Briefly, the method proposed by Santoro et al. [11] involves an alkaline saponification with a 2.0 N ethanolic KOH solution, followed by an extraction with petroleum ether, including several wash cycles; then, the resulting extract was reconstituted in methylene chloride for direct analysis by gas chromatography (GC) and in heptane for further solid phase extraction (SPE) purification of the unsaponifiable fraction. The preferred analytical instrumentation should be gas chromatography coupled with flame ionization detection (GC-FID) as an inexpensive and robust tool available in any laboratory. (ii) To apply the developed method for comparative extraction/migration measurements of DAK from P&B-based food contact samples into food simulants versus some fatty foodstuffs for verification of the specific migration limit of 5 mg/kg food set by German BfR. The selection of the foods to be included in this study was based on two criteria: fatty foods, since they are able to dissolve lipophilic DAK, and fatty foods that can be in contact with in P&B articles. Therefore, we selected olive oil, sunflower oil, croissants, salami sausage, Gouda cheese, and cheddar cheese.

## 2. Results and Discussion

### 2.1. Determination of Dialkylketones (DAK) in Oil and Fatty Foods

#### 2.1.1. Optimization of the Extraction Method

Analysis of foods is often much more complicated than that of food simulants because food is a complex matrix. Interferences in the analysis may occur. The analysis of migrants in foodstuffs mostly involves time-consuming extraction and work-up processes prior to the instrumental analysis. Therefore, here, with the very lipophilic target analytes, DAK, firstly, it was necessary to develop a sample work-up procedure in order to separate the target analytes from the fatty phase of the food. This is a challenging topic due to the similar lipophilicity of the target analytes and the matrix. The saponification method, which removes the fat into the aqueous phase by converting the triacylglycerides into the potassium salts of their corresponding free fatty acids, was chosen as a workable approach. It can be assumed, and was shown, that the ketones are chemically stable under the alkaline saponification conditions and can be recovered by solvent extraction from the unsaponifiable fraction for their quantitative determination.

We used the few pure standards commercially available of dialkylketones (stearone, palmitone) to develop an extraction procedure and gas chromatographic separation method. It can be assumed that all possible DAK of interest have similar structures and physico-chemical properties, and a similar partitioning behavior. Santoro et al. used the same assumption for their method development [11].

For fat/DAK extraction from food, we opted for accelerated solvent extraction (ASE), also known as pressurized fluid extraction (PFE) or pressurized liquid extraction (PLE), since this technique achieves efficient extraction in very short time [18].

Santoro et al. [11] analyzed DAK in samples of vegetable oils. We optimized the extraction and clean-up procedure in order to reduce work and time consumption by removing unnecessary purification steps. From this optimized procedure, a recovery of 85.5%–90% for both DAK from olive oil was obtained.

#### 2.1.2. Method Validation

**Figure 3:**
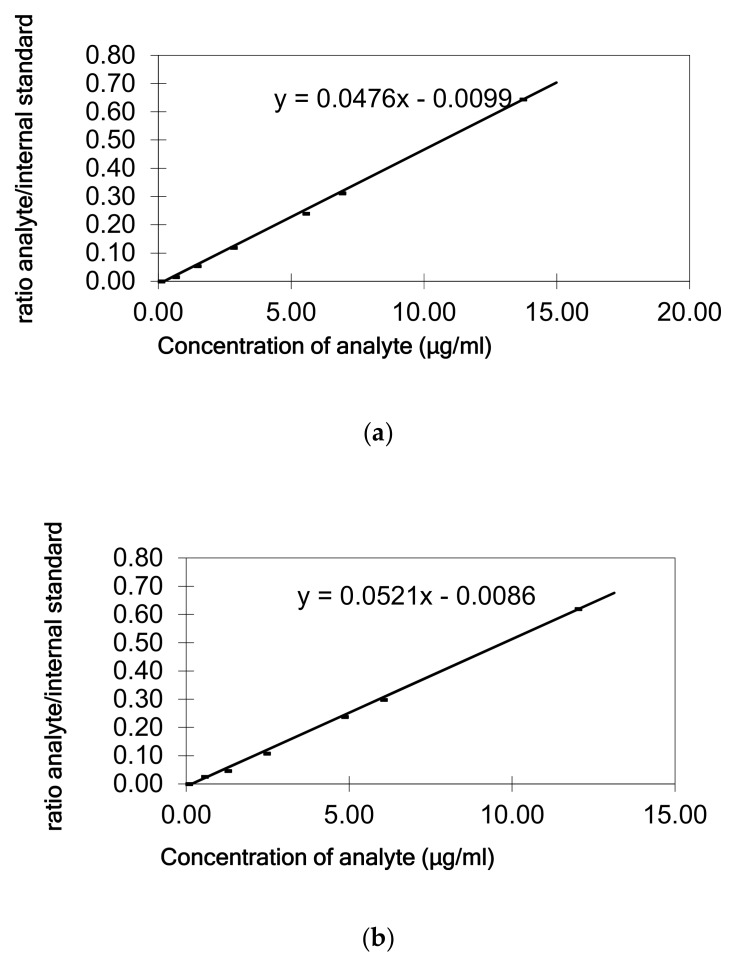
Calibration graphs: (**a**) palmitone in heptane; (**b**) stearone in heptane.

Calibration curves obtained for each standard showed good linearity (Figure 3) in heptane. The regression coefficients were higher than 0.9994. Sufficient sensitivity was achieved for the determination of dialkylketones at the concentration of the SML (5 mg/kg) by this GC-FID method. Limit of quantification (LOQ) was 0.2 µg/mL heptane corresponding to 0.5 µg/g of oil/fat for palmitone and stearone. A good repeatability, also in terms of peak areas and retention times, has been demonstrated, relative standard deviations (RSD %) were equal to 3% for palmitone and 6% for stearone (n = 6).

**Figure 4:**
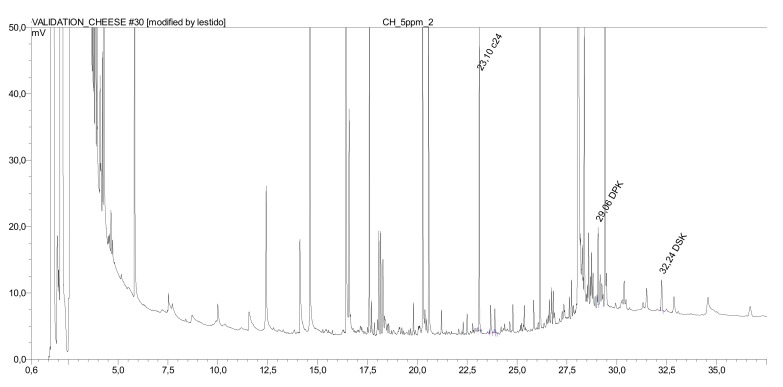
Gas chromatography coupled with flame ionization detection (GC-FID) chromatogram of the extract of cheddar cheese fat spiked with 5 µg/g of dialkylketones: RT 23.10 min tetracosane (IS), RT 29.06 min palmitone, and RT 32.24 min stearone.

Table 1 and Table 2 show the validation data obtained by fortification in the olive oil and the fat of the cheddar cheese, respectively. Five different levels of concentration were tested in duplicate each carrying out the saponification/extraction procedure. The average recovery rates of the target compounds in the oil/fat samples compared to calibration in heptane resulted to be between 81% and 90%. The relative standard deviation (RSD %) at level 2.5 µg/g oil/fat was 3% (n = 6) for both components and both fats. The efficiency whole extraction procedure, including freeze drying and fat extraction with the ASE method, was evaluated in cheddar cheese at two dialkylketone levels, 1 and 5 µg/g (in duplicate), giving recoveries near to 100% (93105%). Figure 4 shows a GC-FID chromatogram of the extract of cheddar cheese fat spiked with 5 µg/g of dialkylketones.

The recovery percentages obtained spiking the fat of croissants, salami, and Gouda cheese samples at two different concentration levels (1 and 5 µg/g duplicate) with a mix standard solution were in the range of 80%–108% (Table 3).

The results demonstrated the potential applicability of this method to quantify the dialkylketones with sufficient sensitivity in the range of, and below, the specific migration limit of 5 mg/kg food. Comparison of the calibrations in the particular food fats with that in heptane showed that calibration in heptane only as a more convenient approach was sufficient.

Compared to the GC-MS method of Santoro et al. (2018) [11], the repeatability at their lowest level of 5 ppm (RSD % > 4.74), sensibility (limit of detection (LODs) > 1.93 ppm and LOQs > 3.15 ppm) and recovery (78%–89%) is improved. The GC-MS method is more selective than the GC-FID method. In case of interferences, the more selective GC-MS method needs to be applied.

### 2.2. Migration of DAK from Paperboard into Oil and Foods versus Solvent Extracts

An overview of the amounts of DAK that were extracted or migrated from the paperboard sample into the solvents, liquid simulants, and fatty foods are given in Table 4.

The extraction of P&B samples with solvents isooctane and dichloromethane was found to be exhaustive after one extraction step. This was verified by a second extraction step from which less than 10% of the first extraction step was found. To say ‘exhaustive’ is not fully correct because the extracted value is linked with the extraction power (swelling potential towards P&B and solubility of DAK) of either of both solvents under the applied time-temperature conditions. Therefore, the extraction values differ, to a certain extent, for both solvents, as can be seen in Table 4.

**Figure 5:**
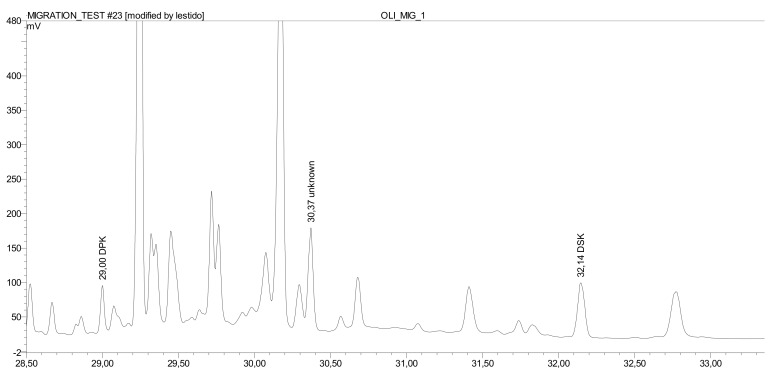
GC-FID chromatogram of the extract of migration in olive oil with palmitone at the RT 29.00 min, another DAK isomer at the RT 30.37 min, and stearone at the RT 32.14 min.

Besides the two isomers, palmitone and stearone, another (mixed) DAK isomer was found and identified by GC-MS. The presence can reasonably be explained and expected due to the fact the applied AKD are technical products consisting of a mixture of chain length isomers (Figure 5). The extraction of the other DAK is present, approximately at the amount of the sum of palmitone and stearone.

Migration into food was calculated based on the quantification results of DAK, the area weight of the paperboard (500 g/m^2^), and applying a surface-to-volume ratio of 6 dm^2^/kg food, according to the European Union cube model. For aspects of compliance, we took into account all DAK detected in the extracts (plus those referred to as “other” in Table 4). The quantification of the “other” DAK was done using the flame ionization detection (FID) response factor of palmitone (it is assumed that all carbon chain lengths show the same mass related FID response).

**Figure 6:**
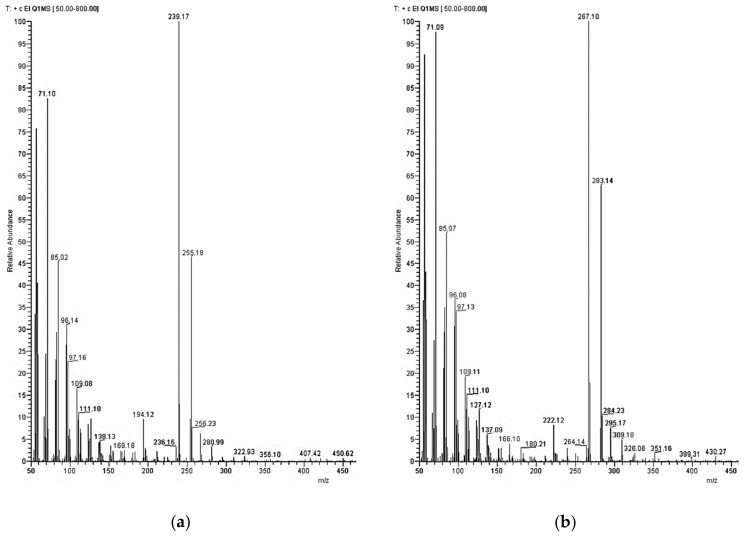
EI mass spectra of palmitone (**a**) and stearone (**b**).

In the case of the croissant sample, an interfering peak was observed at the same retention time as palmitone. Therefore, the sample was analyzed by GC-MS, which allowed separating and quantifying the palmitone in the presence of this interference, since this interference did not show a signal at the selected *m*/*z*. We draw the attention of the reader to this observation to consider application of GC/MS in case of doubts about the identity of the measured GC/FID peaks. As seen in the study of Santoro et al. (2018), the electron ionization mass spectra of dialkylketones show a peculiar pattern characterized by two product ions with a numerical difference between both of 16 u [11]. Figure 6 shows the electron impact ionization (EI) mass spectra of palmitone (a) and stearone (b).

As can be seen from Table 4, there are large differences in the measured migration values between the different solvents and oils versus real fatty food samples. The migration into solvent simulants (isooctane and dichloromethane) must be considered as an extraction and, hence, overestimates by definition the transfer of DAK from paper and board into food. In addition, contact with pure liquid oils due to the intrinsic and penetrative contact has the character of an extraction, and depending on the applied time-temperature conditions, may or may not lead to an exhaustive extraction. We selected sunflower oil to allow measurements at lower temperatures (10 °C) for comparison with the migration into foods under the same t/T-conditions. The values of total DAK obtained by both solvent simulants, and both oils under the applied test conditions, were therefore significantly higher than the specific migration limit of 5 mg/kg set by German BfR. It should be noted that from a toxicological point of view, this SML is relevant for the food and not for solvent extracts.

For the migration measurements with foodstuffs, we selected time-temperature contact conditions, which are representative of somewhat more severe than storage conditions, as occurring in real life. When comparing the migration results obtained for the different fatty food samples, it is striking that these values are by two to three orders of magnitude lower than the solvent/oil extracts. Differences between the food types are only marginal, but tend to depend on factors, such as the fat content of the food and the intensity of the food contact. Higher percentage of fat in the food and higher food contact intensity (pressure and effective contact area of the food sample on the P&B article) will give higher DAK migration. However, in all measured food cases, the DAK migration was far below of the 5 mg/kg SML.

Comparison with other results we found in the literature: The results obtained in the isooctane extraction of the paperboard, expressed as the sum of palmitone and stearone (8 mg/dm^2^), resulted slightly lower, but in the same range, compared to the results obtained under the same conditions for the paperboard in the work of Derra et al. (8.3–11 mg/dm^2^). However, comparing the results obtained in the migration with olive oil, they obtained approximately double under the same conditions (our data: 5 mg/dm^2^ versus 9.3–10 mg/dm^2^) [15]. Of course, such results with solvents and oils depend on the type of P&B material used (level of AKD, production process conditions, sample preparation, and perhaps others); therefore, this comparison has only indicative character. Unfortunately, the authors did not carry out migration measurements into foods.

Comparison with other results we found in the literature: The results obtained in the isooctane extraction of the paperboard, expressed as the sum of palmitone and stearone (8 mg/dm^2^), resulted slightly lower, but in the same range, compared to the results obtained under the same conditions for the paperboard in the work of Derra et al. (8.3–11 mg/dm^2^). However, comparing the results obtained in the migration with olive oil, they obtained approximately double under the same conditions (our data: 5 mg/dm^2^ versus 9.3–10 mg/dm^2^) [15]. Of course, such results with solvents and oils depend on the type of P&B material used (level of AKD, production process conditions, sample preparation, and perhaps others); therefore, this comparison has only indicative character. Unfortunately, the authors did not carry out migration measurements into foods.

Comparison with other results we found in the literature: The results obtained in the isooctane extraction of the paperboard, expressed as the sum of palmitone and stearone (8 mg/dm^2^), resulted slightly lower, but in the same range, compared to the results obtained under the same conditions for the paperboard in the work of Derra et al. (8.3–11 mg/dm^2^). However, comparing the results obtained in the migration with olive oil, they obtained approximately double under the same conditions (our data: 5 mg/dm^2^ versus 9.3–10 mg/dm^2^) [15]. Of course, such results with solvents and oils depend on the type of P&B material used (level of AKD, production process conditions, sample preparation, and perhaps others); therefore, this comparison has only indicative character. Unfortunately, the authors did not carry out migration measurements into foods.

## 3. Materials and Methods

### 3.1. Reagents and Chemicals

All reagents were of analytical grade. Ethanol absolute p.A., ACS, Ph Eur., USP ≤ 99%; n-heptane p.A. ≤ 99%; n-hexane p.A. ≤ 99%; isooctane p.A. ≤ 99.5% and dichloromethane p.A. ≤ 99.8% were obtained from Chemsolute (Th. Geyer GmbH & Co. KG; Renningen, Germany).

The dialkylketone standards 18-pentatriacontanone > 95% (Stearone, DSK; CAS n° 504-53-0, MW 506.94 g/mol) and 16-hentriacontanone > 95% (Palmitone, DPK; CAS n° 502-73-8, MW 450.84 g/mol) were supplied by Tokyo Chemical Industry Deutschland GmbH (TCI; Tokyo, Japan). The internal standard (IS) tetracosane 99% (CAS n° 646-31-1) was purchased from Sigma-Aldrich (St. Louis, MO, USA). Potassium hydroxide pellets p.A. and boiling chips granules were purchased from Merck (Darmstadt, Germany). The cellulose filters for ASE 200 were purchased from Restek (USA).

Stock solutions with a concentration of 1000 mg/L were prepared by dissolving both dialkylketone standard substances (palmitone and stearone) in the solvent n-heptane, after sonication with temperature. A mix standard solution series in the concentration range from 0.2 to 20 mg/L were prepared in n-heptane and were stored at 4 °C until analysis. The tetracosane was added as an internal standard at the 5 µg/mL final concentration. The calibration curve was used for the quantification of the dialkylketones and to verify GC-FID performance.

### 3.2. Food Simulants, Foods, and Paperboard Sample

Olive oil (composed of refined olive oils and virgin olive oils), sunflower oil, salami sausage (fat content 26 g/100 g), young cheddar cheese (fat content 33 g/100 g), young gouda cheese (fat content 27.9 g/100 g), and mini butter croissants (fat content 11 g/100 g) were purchased in local supermarkets (Freising, Germany) and stored in a refrigerator, according to specifications written on the label. The fat content of the foods was taken from the label and was verified by determination of extractable fat, giving results that fit for all samples.

For the migration study, paperboard packaging trays from Mare GmbH, with an area weight of 500 g/m^2^, were used.

### 3.3. Instrumentation

The experiments were performed on a Trace GC 2000 Series ThermoQuest CE Instruments (Austin, TX, USA) gas chromatograph, equipped with a model A200S autosampler (Carlo Erba Instruments, Egelsbach, Germany) and coupled to flame ionization detection.

The GC-MS system used for confirmation was an Agilent 6890 Series gas chromatograph, equipped with a HP 7673 autosampler (Hewlett Packard) and a Thermo Finnigan SSQ mass spectrometer.

The extraction of cheese fat was carried out using an Accelerated Solvent Extractor (ASE) 200 Dionex. A rotavapory vacuum evaporator Büchi rotavapor R-114 coupled to a Büchi vacuum system B-180 and a Büchi waterbath B-480 (Flawil, Switzerland) was used to evaporate the extracts. Freeze-drying of the samples was performed using a Heto Drywinner freeze-dryer model DW 6-85. A water bath with sonication (Bandelin Sonorex, Berlin, Germany) was used to dissolve the standards.

### 3.4. Extraction of DAK from Oil and Foods

#### 3.4.1. Extraction of the Fat in Solid Food Samples

For removal of water, the weighed food samples (except oil) were frozen with liquid nitrogen and freeze-dried for 24 h, at −100 °C and 0.1 mbar.

The extraction of the fat was carried out using an Accelerated Solvent Extractor (ASE). The stainless steel extraction cells of 22 mL were previously cleaned with n-hexane. The lyophilized sample was placed in the stainless steel extraction cell with a filter of cellulose in each side to avoid the blockage of the stainless frit. The extraction cell was placed in the carousel and the samples were extracted under the conditions: 100 bar system pressure, three static cycles, and 15 min-static times/cycle (3 × 15 min static time) at an oven temperature of 120 °C. At the end, the extraction cell was flushed with solvent (flush volume 60%) and purged with nitrogen (purge time 120 s). Hexane was selected as extraction solvent. Finally, the solvent was evaporated using a rotavapor at 60 °C. Residual solvent was removed at 60 °C for 2 h in an oven. Total extractable fat was weighed and determined.

#### 3.4.2. Extraction of the Unsaponifiable Fraction from Fat or Oil

The extraction procedure established in this study was based on the procedure described by Santoro et al. [11] with some simplifying modifications. 2 g of oil or 2 g of the extracted fat (from the first step) was weighed and placed in a flask equipped with a reflux condenser together with 50 mL 2 N potassium hydroxide solution in ethanol 90%. The sample was heated under reflux for 30 min using boiling chips granules to make it boil more calmly. After cooling down, the extract was transferred from the flask into a separatory funnel. The flask was rinsed two times each with 25 mL deionized water, which was transferred (50 mL in total) into the separatory funnel. After addition of 50 mL n-heptane the closed funnel was shaken vigorously. After phase separation, 20 mL of the organic phase was concentrated using a rotatory evaporator to a volume of 2 mL, a volume of 500 µL of 20 mg/L of internal standard (tetracosane) in heptane was added, obtaining 5 mg/L final concentration in the extract. An aliquot of this extract was encapsulated in a glass vial to be analyzed by means of GC-FID or GC-MS. Recovery experiments showed that repeated extraction or additional washing steps were not necessary (recovery from olive oil: 85.5–90%).

### 3.5. Extraction/Migration Tests of Paperboard Sample

Extraction tests were carried out using non-polar solvents, isooctane as food simulant according to the European Standard DIN EN 15519 [19], and additionally, under more severe conditions dichloromethane.

#### 3.5.1. Extraction with Dichloromethane

One gram of the paperboard sample was extracted with 25 mL dichloromethane for 3 days at 40 °C (in duplicate). After this time, 500 µL of the internal standard was added to an aliquot of the extract and analyzed by GC-FID. The quantification was obtained by external calibration in the range of 0.5–12. 5 µg/g for both dialkylketones in n-heptane.

#### 3.5.2. Extraction with Isooctane

One gram of the paperboard sample was extracted with 25 mL isooctane for 1 day at 20 °C (in duplicate). After this time, 500 µL of the internal standard was added to an aliquot of the extract and analyzed by GC-FID. The quantification in the extraction solution was done using the external calibration curve of both dialkylketones in n-heptane.

#### 3.5.3. Migration into Olive Oil

The 10 cm × 10 cm test specimens (1 dm^2^ single side area) of the paperboard sample were cut in small pieces, weighed, and totally immersed in 60 g olive oil for 10 days at 20 °C (in duplicate). Once this time has elapsed, the above described extraction method was applied to obtain the dialkylketones from oil in the unsaponifiable fraction.

#### 3.5.4. Migration into Sunflower Oil

The 10 cm × 10 cm test specimens (1 dm^2^ single side area) of the paperboard sample were cut in small pieces, weighed, and totally immersed in 60 g sunflower oil for one week (7 days) at 10 °C (in duplicate). Once this time has elapsed, the above described extraction method was applied to extract the dialkylketones from oil after saponification.

#### 3.5.5. Migration into Cheddar Cheese, Gouda Cheese, and Salami Sausage

The 1 dm^2^ of the food contact side of the paperboard sample was put in contact with 1 dm^2^ of slices (2–3 mm thickness) of the food samples (cheddar cheese/Gouda cheese/salami). Each experiment was carried out in triplicate. The food slices were pressed on the paperboard to ensure intimate contact. Then these samples were wrapped in aluminum foil in order to eliminate possible contaminations and to minimize a possible loss of the analytes, and stored at 10 °C for 7 days in a controlled climate chamber. The food samples were controlled gravimetrically before and after the migration study, being 25 g of cheddar cheese, 27 g of Gouda cheese, and 8 g of salami. Once this time has elapsed, the paperboard was removed. The fat of the cheese and salami was extracted and the DAK from the fat after saponification, as described above.

#### 3.5.6. Migration with Croissant

The 1 dm^2^ of the food contact side of the paperboard sample was put in contact with 1 dm^2^ of the external side of several croissants cut horizontally (in triplicate). The food slices were pressed on the paperboard to ensure intimate contact. Then these samples were wrapped in aluminum foil and were maintained at room temperature for 24 h (1 day). The sample was also controlled gravimetrically before and after the migration study. Once this time has elapsed, the paperboard was removed, the fat of the croissant was extracted, and then the DAK from the fat after saponification, as described above.

### 3.6. GC-FID Analysis

The analyses were carried out by gas chromatography with flame ionization detection (GC-FID). GC was carried out using a DB-1 capillary column with 30 m length, 0.25 mm inner diameter, and 0.25 µm film thickness. The conditions were as follows: injection temperature 300 °C, injection volume 1 µL in split mode (15 mL/min), flow rate of helium carrier gas 1.5 mL/min. Temperature program: initial temperature 50 °C for 2 min isotherm, ramp to 320 °C with a heating rate of 10 °C/min, and then a final isotherm at 320 °C of 10 min. FID transfer line temperature of 325 °C.

The data were acquired and processed with Chromeleon software (version 6.8). The identification of components was based on comparison of their GC retention time with those obtained for the dialkylketone authentic standards.

Quantification was obtained by external calibration using the ratio of analyte and internal standard area. Olive oil and cheddar cheese calibration was carried out by adding the palmitone and stearone standards to the oil or the extracted fat and performing the whole saponification/extraction procedure. The other foods were quantified using calibration in heptane.

### 3.7. GC-MS Analysis

GC was carried out using an Optima-5MS column with 30 m length, 0.25 mm inner diameter, and 0.25 µm film thickness. The conditions were as follow: injection temperature 320 °C, injection volume 2 µL in split mode (1:10), flow rate of helium carrier gas 1.5 mL/min. Temperature program: initial temperature 50 °C for 2 min isotherm, then heating rate of 10 °C/min, and a final isotherm at 340 °C of 30 min. Mass detector conditions: transfer line temperature 320 °C and EI ionization mode (70 eV) with selective ion monitoring (SIM). The SIM masses selected were *m*/*z* 239 and 255 for palmitone and *m*/*z* 267 and 283 for stearone.

The data were acquired and processed with Xcalibur software. The identification of components was based on comparison of their GC retention time with those obtained for the dialkylketone authentic standards and using the National Institutes of Standards and Technology (NIST) mass spectra library. For quantification, the sum of both mass fragments per substance was used.

### 3.8. Method Validation

The analytical characteristics of the developed method for these dialkylketones were evaluated, including linearity, sensitivity, precision, and trueness.

Linearity of the detector response was verified with standard solutions in heptane at six concentrations (0.2, 0.5, 1.0, 2.0, 2.5, and 5.0 µg/mL heptane corresponding to 0.5, 1.25, 2.5, 5, 6.25, and 12.5 µg/g of fat/oil). As internal standard tetracosane (5 µg/mL) was used for quantitative analysis in order to compensate instrumental variability of GC-FID. Calibration curves were constructed by plotting the standard concentration versus the peak area/internal standard area ratio obtained from GC-FID.

The quantification of dialkylketones in olive oil and the fat of the cheddar cheese were done using the calibration curve in the oil or cheese fat, respectively. Heptane standard solutions containing five different concentration levels of palmitone and stearone (0.5, 1.25, 2.5, 5, and 12.5 µg/g fat) were added to the oil/fat. Each level was done in duplicate and the sample without spiking was analyzed too. The quantification was based on calibration curves generated by plotting the peak area/internal standard ratio versus the amount spiked of each standard. In parallel standards of palmitone and stearone directly in heptane were measured, and calibration curves calculated. The trueness of the extraction and quantification method developed has been established in terms of recovery (by comparing the slopes of the calibration line obtained from the spiked olive oil/fat cheddar cheese and the calibration in n-heptane). The efficiency of the cheddar cheese fat extraction was also evaluated through recovery studies from spiked cheddar cheese samples with known amounts of dialkylketones (1 and 5 µg/g) in duplicate, before the cheese was subject to the freeze-drying process. Extraction and GC analysis were conducted using the same sample procedure.

Recoveries of the other foods (croissants, salami, and Gouda cheese) were determined by fortifying the extracted fat with 500 µL of mix standard solutions at two concentration levels (corresponding to 1 and 5 µg/g fat) in duplicate.

The sensitivity of the method was studied by determining the limit of detection (LOD) and limit of quantification (LOQ) for individual compounds in standard solutions taking into account the noise in the chromatographic analyses. The examination of a blank was always carried out in parallel. The limit of detection was considered as the lowest concentration, which provides a signal-to-noise ratio of 3, while the limit of quantification was considered as a signal-to-noise ratio of 10.

The precision of the extraction and measurement procedure was determined as repeatability for both standard compounds by extraction two spiked olive oil, and the cheddar cheese fat samples, at 3 different days, at a concentration of 2.5 µg/g (n = 6) and expressed as relative standard deviations (RSDs %).

## 4. Conclusions

A convenient and cost-effective method was developed and validated in this study for the extraction and determination of dialkylketones in edible oils and real fatty food samples using the gas chromatography technique with flame ionization detection (GC-FID). In addition, a gas chromatography with mass spectrometry (GC-MS) method was established for confirmation and quantification purposes when there was any interference in the food sample.

Based on this method, extraction tests of a paper-based food contact article with conventional food simulant solvent isooctane and extraction solvent dichloromethane, as well as extractive migration into edible oils (olive oil, sunflower oil) were carried out and compared with migration tests into real fatty food samples (croissants, salami, Gouda cheese, and cheddar cheese). As a result, it was found that the simulating tests, including the edible oil extraction tests, gave migration values exceeding the specific migration limit (SML) of 5 mg/kg, established by BfR largely. On the other hand, the migration results with the food samples were two to three orders of magnitude lower, and in any case, largely below the SML.

Our results, with the example of DAK as migrants, indicate one of the weaknesses in P&B migration testing, where extraction tests are dominating in opposite to migration testing of plastics: simulation of migration from P&B articles with conventional solvent based food simulants can be heavily misleading when the intention is to verify a given SML, or to provide data for exposure estimation. Clearly, isooctane extractions of P&B samples for verifying compliance with the DAK SML give ‘migration’ values far above realistic values obtainable when testing migration into foods.

When comparing the results obtained between the different fatty food samples after the contact with paperboard, it appears—although this is not proven and still needs further studies—that the extent of the migration of the dialkylketones present in the paper and board-based food packaging may depend on the fat content of the food, and on the intensity of the contact between food and P&B material.

We acknowledge that our studies are not fully comprehensive, and see the need for further studies with more P&B materials and other migrants, as well as other foods, including kinetic studies. However, the results presented here for DAK confirm, to a large degree, what one can expect for migration of P&B constituents into foods: in many cases such migration values will be lower and even considerably lower than those results obtained by solvent extraction tests. Clearly, the level of migration and, hence, the closeness of the results from the extraction versus into food migration will depend on the chemical nature and volatility of the migrant. Future research in this topic will further contribute to a more exposure-orientated assessment of migration of paper additives and their components into foodstuffs.

## Figures and Tables

**Table 1:** Calibration curve in olive oil.

	Slope	Intercept	Correlation Coefficient (R^2^)	LOQ (µg/g of Oil)	Recovery (%)	Repeatability (RSD) %) (n = 6)
**Palmitone**	0.0407	0.1191	0.9998	0.5	85.6 ± 1.0	3
**Stearone**	0.0470	−0.0039	0.9997	0.5	90.3 ± 1.2	3

**Table 2:** Calibration curve in the fat of the cheddar cheese.

	Slope	Intercept	Correlation Coefficient (R^2^)	LOQ (µg/g of Oil)	Recovery (%)	Repeatability (RSD) %) (n=6)
**Palmitone**	0.0394	0.0539	0.9998	0.5	86.1 ± 0.87	3
**Stearone**	0.0410	−0.0004	0.9999	0.5	81.1 ± 0.42	3

**Table 3:** Recovery percentages obtained spiking the fat of croissants, salami, and Gouda cheese samples.

Spiked Level	Croissant		Salami		Gouda cheese	
	Palmitone *	Stearone	Palmitone	Stearone	Palmitone	Stearone
**1 µg/g**	95	102	80	96	80	90
**5 µg/g**	108	82	83	82	83	80

* via gas chromatography with mass spectrometry (GC-MS).

**Table 4:** Results of solvent extraction and migration test of the dialkylketones (DAK) from paper and board (P&B) sample into food (mg/6 dm^2^).

	Isooctane (1 day/25 °C)	DCM (3 day/40 °C)	Olive Oil (10 day/20 °C)	Sunflower Oil (7 day/10 °C)	Cheddar Cheese (7 day/10 °C)	Gouda Cheese (7 day/10 °C)	Salami (7 day/10 °C)	Croissant (1 day/RT)
**Palmitone**	15	18	10	8	0.040	0.031	0.012	0.007
**Stearone**	33	44	20	15	0.060	0.053	0.031	0.011
**Other DAK**	47	86	27	22	nd	nd	nd	nd
**Total DAK**	95	148	57	45	0.1	0.084	0.043	0.018

* nd: not determined; DCM: dichloromethane

## References

[1] Confederation of European Paper Industries (CEPI), Industry Guideline for the Compliance of Paper & Board Materials and Articles for Food Contact (2012). http://www.cepi.org/publication/industry-guideline-compliance-paper-board-materials-and-articles-food-contact-issue-2.

[2] Varshoei A., Javid E., Rahmaninia M., Rahmany F. (2014). The Performance of Alkylketene Dimer (AKD) for the Internal Sizing of Recycled OCC Pulp. Lignocellul. J..

[3] Kumar S., Chauhan V.S., Chakrabarti S.K. (2016). Separation and analysis techniques for bound and unbound alkyl ketene dimer (AKD) in paper: A review. Arab. J. Chem..

[4] Seo W.S., Cho N.S. (2005). Effect of water content on cellulose/AKD reaction. Appita J..

[5] Asakura K., Iwamoto M., Isogai A. (2005). Effects of fatty acid components present in AKD wax on emulsion stability and paper sizing performance. J. Wood Chem. Technol..

[6] Bildik A.E., Hubbe M.A., Gurboy K.B. (2016). Alkyl ketene dimer (AKD) sizing of paper under simplified treatment conditions. Tappi J..

[7] Karademir A., Varlibas H., Karahan S., Aydemir C. (2011). Pre-treatment of cellulose fibres with some chemicals for effective sizing. O Papel.

[8] Lindström T., Söderberg G. (1986). On the mechanism of sizing with alkylketene dimers, 1: Studies on the amount of alkylketene dimer required for sizing different pulps [AKD, reaction mechanism]. Nord. Pulp Paper Res. J..

[9] Stocchi A., Iacopini M. (2019). Food contact paper and paperboard: Examples of gas and liquid chromatography determinations. Food Contact Materials Analysis.

[10] Bradley E.L., House A., Day J.S., Driffield M., Hutton S. (2010). Biobased Materials Used in Food Contact Applications: An Assessment of the Migration Potential.

[11] Santoro V., Baiocchi C., Dal Bello F., Gastaldi D., Aigotti R., Zorzi M., Pellegrino A., Forte E., Romaniello F., Magni M. (2018). Formation of by-products during chemical interesterification of lipids. Detection and characterization of dialkyl ketones by non-aqueous reversed-phase liquid chromatography-high resolution mass spectrometry and gas chromatography-mass spectrometry. J. Chromatogr. A.

[12] ChemSpider: Stearone. http://www.chemspider.com.

[13] BfR Recommendation XXXVI (2017). Paper and board for food contact.

[14] U.S. Food and Drug Administration (FDA) (2018). Code of Federal Regulations (CFR), Title 21, Part 176.120—Indirect Food Additives: Paper and Paperboard Components.

[15] Derra R., Jung T. Evaluation of the Transfer of Dialkylketones Into Foodstuffs. Proceedings of the 6th International Symposium on Food Packaging: Scientific Developments Supporting Safety and Innovation.

[16] Mariani C., Bellan G. (2011). Individuazione di oli e grassi interesterificati con metodologie diverse. Riv. Ital. Sostanze Grasse.

[17] Rohm H., Schäper C., Zahn S. (2018). Interesterified fats in chocolate and bakery products: A concise review. LWT Food Sci. Technol..

[18] Kang J.H., Kim S., Moon B. (2016). Optimization by response surface methodology of lutein recovery from paprika leaves using accelerated solvent extraction. Food Chem..

[19] (2020). DIN EN 15519 Paper and Board Intended to Come into Contact with Foodstuffs—Preparation of an Organic Solvent Extract.

